# lncRNA PCAT14 Is a Diagnostic Marker for Prostate Cancer and Is Associated with Immune Cell Infiltration

**DOI:** 10.1155/2021/9494619

**Published:** 2021-12-31

**Authors:** Yunkun Yan, Jianjun Liu, Zhijian Xu, Mushi Ye, Jianchang Li

**Affiliations:** Department of Urology, Affiliated Hospital of Guangdong Medical University, Zhanjiang, Guangdong, China

## Abstract

**Objective:**

To investigate the relationship between the long noncoding RNA (lncRNA) *Prostate cancer-associated transcription factors 14* (*PCAT14*) and the clinical characteristics of prostate cancer and immune cell infiltration.

**Methods:**

The relationship between *PCAT14* expression and the clinicopathological characteristics of prostate cancer was analyzed based on The Cancer Genome Atlas (TCGA) database. Receiver operating characteristic (ROC) curves were used to evaluate the value of *PCAT14* as a diagnostic marker for prostate cancer. The relationship between *PCAT14* and immune cell infiltration was analyzed to explore the effect of *PCAT14* on the immune-related functions of prostate cancer.

**Results:**

The ROC curve showed that *PCAT14* had a significant diagnostic ability (area under curve = 0.818) for prostate cancer. A reduced expression of *PCAT14* in prostate cancer was related to T stage, N stage, primary therapy outcome, residual tumor, Gleason score, and age. The expression of *PCAT14* was independently associated with the progression-free interval in prostate cancer patients. The infiltration of immune cells in prostate cancer showed a significant negative correlation between the expression of *PCAT14* and plasmacytoid dendritic cells, activated dendritic cells, regulatory T cells, and neutrophils.

**Conclusions:**

*PCAT14* is highly expressed in prostate cancer and is expected to be a diagnostic marker. *PCAT14* might promote the development of prostate cancer through chemokines, antimicrobials, and cytokines that affect the infiltration of immune cells.

## 1. Introduction

Prostate cancer is the second leading cause of cancer-related deaths in men, according to the 2018 global cancer incidence and mortality statistics [1]. Prostate-specific antigen (PSA) is used as the main marker for prostate cancer screening, diagnosis, and prognosis. Nevertheless, PSA alone as a single marker still has great limitations in diagnosing and determining prostate cancer prognosis. Therefore, it is necessary to continue the search for more potentially effective markers for the diagnosis and prognosis of prostate cancer. In addition, the treatment of prostate cancer has made some progress, but it is still not satisfactory. Therefore, further studies on the pathogenesis of prostate cancer are still needed to provide more clues for the treatment of prostate cancer.

Long noncoding RNAs (lncRNAs) are long RNA transcripts of over 200 nucleotides in length, but they do not encode proteins [2]. Several studies have found that lncRNAs play important roles in various types of cancer [3–7]. At present, it is generally agreed that lncRNAs have a strong application prospect as tumor diagnostic markers and might eventually be targeted for therapy [8]. Shukla et al. [9] identified the lncRNA *Prostate cancer-associated transcription factors 14* (*PCAT14*) in prostate cancer and that the overexpression of *PCAT14* inhibited the invasion of prostate cancer cells and was associated with a good prognosis of prostate cancer. Still, there are few reports on the association between *PCAT14* and prostate cancer, the relationship between *PCAT14* and the clinical indicators of prostate cancer, and the relationship between *PCAT14* and immune cell infiltration in prostate cancer. Indeed, the tumor microenvironment and immune cell infiltration are now recognized to play crucial roles in the development and prognosis of solid cancers [10–14].

Therefore, this study is aimed at investigating the relationship between the LncRNA *PCAT14* and the clinical characteristics of prostate cancer and immune cell infiltration. This study performed a detailed comparative analysis of lncRNA *PCAT14* and prostate cancer-related clinical indicators (age, race, diagnostic validity, tumor-node-metastasis (TNM) staging, initial treatment outcome, residual tumor, PSA, Gleason score, and overall survival). A detailed comparative analysis was conducted to analyze the correlation between *PCAT14* and prostate cancer disease progression. In addition, we attempted to explore the correlation between *PCAT14* and immune cell infiltration in the prostate cancer tumor microenvironment and analyzed the immunological pathways closely related to prostate cancer. The results might provide a scientific basis for future research on prostate cancer immunotherapy.

## 2. Materials and Methods

### 2.1. The Downloading of the RNA Sequencing (RNAseq) Data and Analysis of *PCAT14* Expression in Prostate Cancer

RNAseq data and clinical information were downloaded from the PRAD project in The Cancer Genome Atlas (TCGA) database. The RNAseq data were converted into the transcripts per million reads (TPM) format, and log2 conversion was performed. The data were from 499 prostate cancer samples and 52 adjacent normal tissue samples. The significance of expression level was as follows: ns, *p* ≥ 0.05; ∗, *p* < 0.05; ∗∗, *p* < 0.01; and ∗∗∗, *p* < 0.001.

### 2.2. Correlation Analysis of PCAT14 and Immune Invasion in Prostate Cancer

The single-sample Gene Set Enrichment Analysis (SSGSEA) method in the Gene Set Variation Analysis (GSVA) package (1.34.0 version) of R (3.6.3 version) was used to analyze the infiltration of immune cells in prostate cancer. The markers and classification of 24 kinds of immune cells were obtained from a published paper [11]. The correlation between *PCAT14* and the level of immune cell infiltration in prostate cancer was determined using the Spearman and Wilcoxon rank-sum tests. Immlnc is a tool that can be used to study the immune function of lncRNAs in different cancer types [15]. This web tool can be used to inquire online about the relationship between lncRNAs and immune cell infiltration in specific cancers. At the same time, the tool supports queries of lncRNAs' potential immune-related biological pathways.

### 2.3. Statistical Methods

All analyses were performed using R (v.3.6.3). The correlations between *PCAT14* and clinical indicators were analyzed by the Wilcoxon's rank-sum test, chi-square test, Fisher's exact test, and logistic regression. The Cox proportional risk model was used to assess the association between clinical indicators and progression-free interval (PFI). A *P* value < 0.05 was considered statistically significant.

## 3. Results

### 3.1. PCAT14 Can Be Used as a Potential Marker for Diagnosis of Prostate Cancer

**Figure 1:**
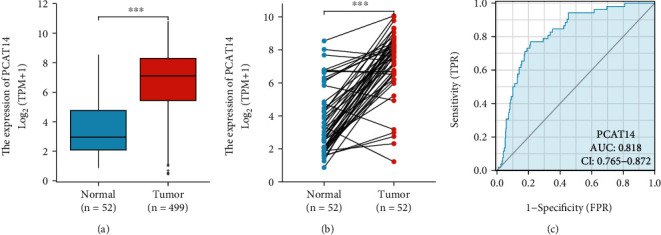
Expression and diagnostic efficacy of PCAT14 in prostate cancer. (a) Comparison of the expression of *PCAT14* in 499 prostate cancer samples and 52 paracancer normal tissue samples. The results showed that *PCAT14* was highly expressed in prostate cancer tissues. (b) Comparison of the expression of *PCAT14* in 52 prostate cancer tissues and its matching paracancer tissues. The results showed that *PCAT14* was highly expressed in prostate cancer tissues. (c) Received operating characteristics (ROC) curve analysis showed that the area under the curve (AUC) of *PCAT14* was 0.818, indicating that *PCAT14* has a significant diagnostic ability.

We analyzed the expression of *PCAT14* in 499 prostate cancer samples and 52 adjacent normal tissue samples, and the results showed that *PCAT14* was highly expressed in prostate cancer tissues (Figure 1(a)). We also analyzed the expression of *PCAT14* in 52 prostate cancer tissues and their matched adjacent tissues. The results also showed that *PCAT14* was highly expressed in prostate cancer tissues (Figure 1(b)). In addition, receiver operating characteristic (ROC) curves were used to analyze the diagnostic validity of the *PCAT14* expression level. The area under the curve (AUC) of *PCAT14* was 0.818, indicating that *PCAT14* can be used as an ideal biomarker for diagnosing prostate cancer (Figure 1(c)).

### 3.2. Correlation between PCAT14 and Clinical Indicators of Patients

**Figure 2:**
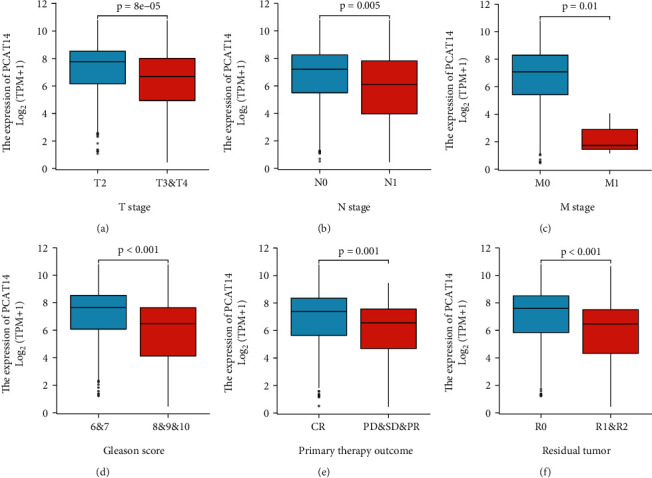
Relationship between *PCAT14* expression and clinically relevant indicators in prostate cancer. (a) Compared with T2 stage prostate cancer, *PCAT14* was lowly expressed in T3-4 stage prostate cancer tissues. (b) Compared with N0 stage prostate cancer, *PCAT14* was lowly expressed in N1 stage prostate cancer tissues. (c) Compared with M0 stage prostate cancer, *PCAT14* was lowly expressed in M1 stage prostate cancer tissue. (d) Compared with the prostate cancer group with Gleason score = 6-7, *PCAT14* was lowly expressed in prostate cancer tissue with Gleason score = 8-10. (e) *PCAT14* was lowly expressed in prostate cancer tissues with the initial treatment outcome of progressive disease, stable disease, and partial response compared with prostate cancer tissues with initial treatment outcome of complete response. (f) *PCAT14* was lowly expressed in R1&2 prostate cancer tissue compared with prostate cancer in R0 prostate cancer tissue.

According to the average relative expression level of *PCAT14*, prostate cancer patients were divided into two groups: the high *PCAT14* expression group (*n* = 250) and the low *PCAT14* expression group (*n* = 249). Then, the expression levels of *PCAT14* and the correlations with clinical indicators were analyzed. A reduced expression of *PCAT14* in prostate cancer was related to T stage (*P* = 0.005), N stage (*P* = 0.009), primary therapy outcome (*P* = 0.022), residual tumor (*P* < 0.001), Gleason score (*P* < 0.001), and age (*P* = 0.020) (Table 1, Figure 2).

### 3.3. Cox Univariable and Multivariable Analysis of Prognostic Factors in Prostate Cancer

Table 2 shows the results of the Cox univariable and multivariable analyses of PFI in prostate cancer patients. In the Cox univariable regression model, the variables with *P* < 0.01 were T stage (*P* < 0.001), N stage (*P* = 0.007), primary treatment outcome (*P* < 0.001), PSA (*P* < 0.001), and the Gleason score (*P* < 0.001). The multivariable analysis showed that the primary treatment outcome (*P* < 0.001) and Gleason score (*P* < 0.001) were independent prognostic factors for PFS in prostate cancer patients.

### 3.4. Relationship between *PCAT14* Expression and Immune Cell Infiltration in Prostate Cancer

**Figure 3:**
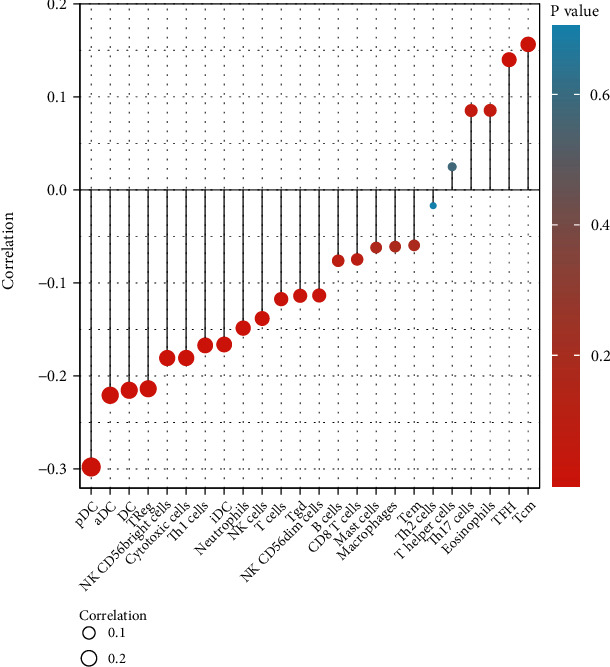
Forest plot showed that the expression of *PCAT14* in the tumor microenvironment of prostate cancer was negatively correlated with the infiltration of plasmacytoid dendritic cells, activated dendritic cells, regulatory T cells, and neutrophils.

**Figure 4:**
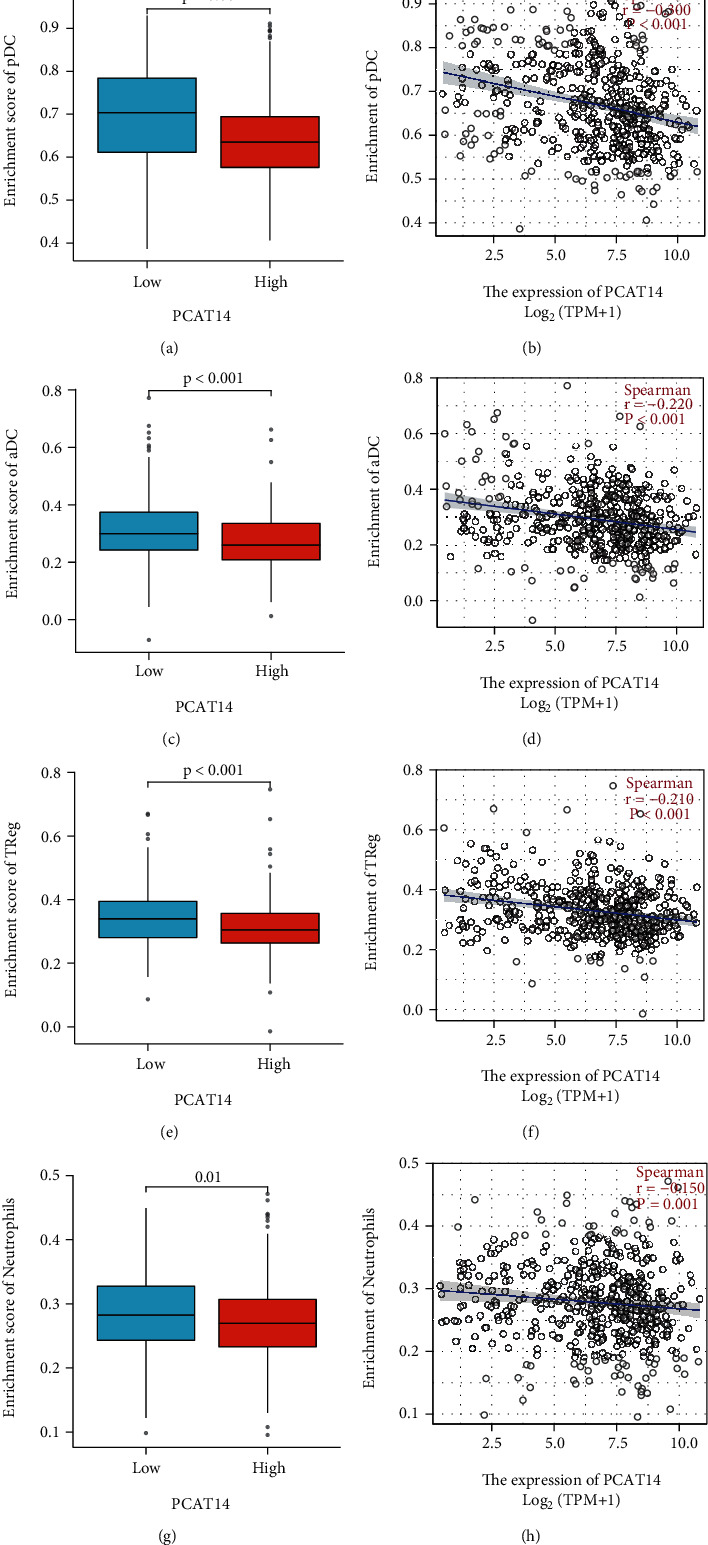
Correlation analysis of *PCAT14* expression level with pDC, aDC, Treg, and neutrophil infiltration. (a) The Wilcoxon rank-sum test analysis showed that the plasmacytoid dendritic cell (pDC) infiltration level of the high *PCAT14* expression group was low, and the pDC infiltration level of the low *PCAT14* expression group was high, and the difference was statistically significant. (b) The Spearman correlation analysis showed that the expression level of *PCAT14* was negatively correlated with the infiltration of pDC. (c) The Wilcoxon rank-sum test analysis showed that the activated dendritic cells (aDC) infiltration level in the high *PCAT14* expression group was low, and the aDC infiltration level in the low *PCAT14* expression group was high, and the difference was statistically significant. (d) The Spearman correlation analysis showed that the expression level of *PCAT14* was negatively correlated with the infiltration of aDC. (e) The Wilcoxon rank-sum test analysis showed that the level of regulatory T cell (Treg) infiltration in the high *PCAT14* expression group was low, the Treg infiltration level in the low *PCAT14* expression group was high, and the difference was statistically significant. (f) The Spearman correlation analysis showed that the expression level of *PCAT14* was negatively correlated with Treg infiltration. (g) The Wilcoxon rank-sum test analysis showed that the level of neutrophil infiltration in the high *PCAT14* expression group was low, the neutrophil infiltration level in the low *PCAT14* expression group was high, and the difference was statistically significant. (h) The Spearman correlation analysis showed that the expression level of *PCAT14* was negatively correlated with the infiltration of neutrophils.

We analyzed the relationship between *PCAT14* expression and immune cell infiltration in prostate cancer using SSGSEA. The results showed that *PCAT14* expression was negatively correlated with the infiltration of plasmacytoid dendritic cells (pDC), activated dendritic cells (aDC), regulatory T cells (Tregs), and neutrophils (Figures 3 and 4).

We verified the correlation between *PCAT14* and immune cell infiltration using the Immlnc database. The results also showed that *PCAT14* was negatively correlated with neutrophils and dendritic cells (*P* < 0.05) (Table 3). In addition, we analyzed the relationship between *PCAT14* and immune-related pathways in prostate cancer by GSEA. The results showed that the immune pathways related to *PCAT14* included chemokines, antimicrobials, and cytokines (*P* < 0.05) (Table 4).

## 4. Discussion

At present, scholars have explored the relationship between lncRNAs and prostate cancer. He et al. [16] identified regulatory relationships among lncRNAs, miRNAs, and mRNAs that might be involved in the pathogenesis of prostate cancer. Still, few studies have reported the association between *PCAT14* and prostate cancer. The research on the tumor microenvironment is also a hot topic at present. Immune cells, as important members of the tumor microenvironment, are closely related to the tumor [17]. At present, the relationship between *PCAT14* and immune cell infiltration in prostate cancer is still unclear.

This study first analyzed the expression of the lncRNA *PCAT14* in prostate cancer and its relationship with prostate cancer prognosis. The results showed that *PCAT14* was significantly overexpressed in prostate cancer. In addition, the ROC curve analysis showed that *PCAT14* could be a biomarker for diagnosing prostate cancer. *PCAT14*, a new potential diagnostic marker for prostate cancer, could make up for the nonincreased PSA levels in diagnosing prostate cancer.

At present, several studies have shown that the infiltration of immune cells in prostate cancer promotes the occurrence and development of prostate cancer [18]. Jiang et al. [19] found that infiltrating immune cells could promote the proliferation, invasion, and distant metastasis of tumor stem cells in prostate and breast cancers. Gannon et al. [20] found that androgen deprivation therapy could regulate the infiltration of immune cells and proved that NK cells had a protective effect on tumor progression, while macrophages were beneficial to the progression of advanced prostate cancer. The highlight of our study was to reveal the relationship between *PCAT14* expression and a variety of immune cells infiltrating prostate cancer. Our results showed that the expression of *PCAT14* was negatively correlated with the infiltration level of pDC, aDC, Tregs, and neutrophils in prostate cancer. Since dendritic cells (DC) [12] and neutrophils [13] mainly play an antitumor role in the tumor microenvironment, *PCAT14* might promote the progression of prostate cancer by inhibiting the antitumor effect of DC and neutrophils. We also observed a negative correlation between *PCAT14* expression and the infiltration level of Tregs in prostate cancer. Currently, it is widely believed that Tregs generally inhibit the immune response of tumors [21]. Still, some authors have proposed that Treg cells play a double-edged role in solid tumors [22]. Further experiments are needed to confirm the association and analyze the specific biological significance of the various immune cell subtypes in prostate cancer progression. In addition, we analyzed the relationship between *PCAT14* and immune-related pathways in prostate cancer by GSEA. The results showed that the immune pathways related to *PCAT14* included chemokines, antimicrobials, and cytokines. Several studies suggested that cytokines and chemokines, along with their receptors and signaling axes, are important factors driving prostate cancer metastasis [10–14].

This study provides a scientific basis for further research on the pathogenesis of the lncRNA *PCAT14* in prostate cancer. This study showed that *PCAT14* has significant diagnostic ability in prostate cancer and might be expected to become an effective screening and diagnostic marker for prostate cancer. This research also found that the expression of *PCAT14* is negatively correlated with the infiltration of pDC, aDC, Tregs, and neutrophils in the tumor microenvironment of prostate cancer. *PCAT14* might affect the infiltration of immune cells through chemokines, antibacterial agents, and cytokines. Such immunological pathways promote the pathogenesis and progression of prostate cancer [10–14], providing a theoretical basis for future research on prostate cancer immunotherapy. *In vitro* and *in vivo* studies are necessary to confirm these roles and explore potential interventions based on *PCAT14*.

In conclusion, we observed an increased expression of *PCAT14* in prostate cancer tissue. *PCAT14* might be promising as a diagnostic marker for prostate cancer. In addition, *PCAT14* probably influences the infiltration of immune cells through chemokines, antimicrobials, and cytokines, thus promoting prostate cancer development.

## Figures and Tables

**Table 1:** Correlation between *PCAT14* and clinical indicators of patients.

	Low expression of *PCAT14*	High expression of *PCAT14*	*P*
*n*	249	250	
T stage, *n* (%)			0.005
T2	76 (15.4%)	113 (23%)	
T3	160 (32.5%)	132 (26.8%)	
T4	7 (1.4%)	4 (0.8%)	
N stage, *n* (%)			0.009
N0	169 (39.7%)	178 (41.8%)	
N1	52 (12.2%)	27 (6.3%)	
M stage, *n* (%)			0.249
M0	230 (50.2%)	225 (49.1%)	
M1	3 (0.7%)	0 (0%)	
Primary therapy outcome, *n* (%)			0.022
PD	16 (3.7%)	12 (2.7%)	
SD	18 (4.1%)	11 (2.5%)	
PR	27 (6.2%)	13 (3%)	
CR	156 (35.6%)	185 (42.2%)	
Race, *n* (%)			0.195
Asian	9 (1.9%)	3 (0.6%)	
Black or African American	30 (6.2%)	27 (5.6%)	
White	204 (42.1%)	211 (43.6%)	
Age, *n* (%)			0.342
≤60	106 (21.2%)	118 (23.6%)	
>60	143 (28.7%)	132 (26.5%)	
Residual tumor, n (%)			<0.001
R0	133 (28.4%)	182 (38.9%)	
R1	100 (21.4%)	48 (10.3%)	
R2	1 (0.2%)	4 (0.9%)	
PSA (ng/ml), *n* (%)			0.347
<4	199 (45%)	216 (48.9%)	
≥4	16 (3.6%)	11 (2.5%)	
Gleason score, *n* (%)			< 0.001
6	16 (3.2%)	30 (6%)	
7	103 (20.6%)	144 (28.9%)	
8	34 (6.8%)	30 (6%)	
9	92 (18.4%)	46 (9.2%)	
10	4 (0.8%)	0 (0%)	
OS event, *n* (%)			0.339
Alive	246 (49.3%)	243 (48.7%)	
Dead	3 (0.6%)	7 (1.4%)	
Age, mean ± SD	61.74 ± 6.48	60.32 ± 7.08	0.020
PSA (ng/ml), mean ± SD	2.79 ± 22.41	0.76 ± 3.34	0.179

PD: progressive disease; SD: stable disease; PR: partial response; CR: complete response; PSA: prostate-specific antigen; OS: overall survival.

**Table 2:** Cox univariable and multivariable analysis of PFI in prostate cancer patients.

	Total (*n*)	Univariable analysis		Multivariable analysis	
		Hazard ratio (95% CI)	*P* value	Hazard ratio (95% CI)	P value
T stage (T3&T4 vs. T2)	492	3.785 (2.140-6.693)	<0.001	1.473 (0.710-3.059)	0.299
N stage (N1 vs. N0)	426	1.946 (1.202-3.150)	0.007	0.861 (0.500-1.483)	0.589
M stage (M1 vs. M0)	458	3.566 (0.494-25.753)	0.208		
Primary therapy outcome (PD&SD&PR vs. CR)	438	6.627 (4.337-10.126)	<0.001	3.576 (2.054-6.226)	<0.001
Race (Asian & Black or African American vs. White)	484	0.751 (0.417-1.352)	0.339		
Age (>60 vs. ≤60)	499	1.302 (0.863-1.963)	0.208		
Residual tumor (R1&R2 vs. R0)	468	2.365 (1.566-3.570)	<0.001	1.017 (0.598-1.728)	0.951
PSA (ng/ml) (≥4 vs. <4)	442	4.196 (2.095-8.405)	<0.001	1.650 (0.743-3.667)	0.219
Gleason score (8-10 vs. 6-7)	499	4.675 (2.957-7.391)	<0.001	2.901 (1.603-5.253)	<0.001

CI: confidence interval; PSA: prostate-specific antigen.

**Table 3:** Correlation between *PCAT14* and immune cells in prostate cancer.

Cancer	lncRNA ID	lncRNA symbol	Immune cell	*P* value
PRAD	ENSG00000280623	PCAT14	Dendritic	<0.001
PRAD	ENSG00000280623	PCAT14	Neutrophil	< 0.001

lncRNA: long noncoding RNA; PRAD: prostate adenocarcinoma.

**Table 4:** Correlation between *PCAT14* and immune-related pathways in prostate cancer.

Cancer	lncRNA ID	lncRNA symbol	Immune pathway	*P* value^a^	ES^b^
PRAD	ENSG00000280623	PCAT14	Chemokines	0.003384	0.488026
PRAD	ENSG00000280623	PCAT14	Antimicrobials	0.025140	0.327294
PRAD	ENSG00000280623	PCAT14	Cytokines	0.028011	0.334931
PRAD	ENSG00000280623	PCAT14	TNF family members	0.090566	0.658357
PRAD	ENSG00000280623	PCAT14	Interferon receptor	0.206693	0.791169
PRAD	ENSG00000280623	PCAT14	Interleukins receptor	0.164966	0.440330

^a^ *P* value: the *P* values for GSEA analysis; ^b^ES: enrichment scores; lncRNA: long noncoding RNA; ES: enrichment score; PRAD: prostate adenocarcinoma.

## Data Availability

We did not create any data, and all the results we analyzed in our paper were based data from public database. So we do not have any data that could be uploaded to a repository.
